# “Why Do They Need to Check Me?” Patient Participation Through eHealth and the Doctor-Patient Relationship: Qualitative Study

**DOI:** 10.2196/jmir.8444

**Published:** 2018-01-15

**Authors:** Christiane Grünloh, Gunilla Myreteg, Åsa Cajander, Hanife Rexhepi

**Affiliations:** ^1^ School of Computer Science and Communication KTH Royal Institute of Technology Stockholm Sweden; ^2^ Institute of Informatics Technische Hochschule Köln University of Applied Sciences Gummersbach Germany; ^3^ Department of Business Studies Uppsala University Uppsala Sweden; ^4^ Department of Information Technology Uppsala University Uppsala Sweden; ^5^ School of Informatics University of Skövde Skövde Sweden

**Keywords:** patient accessible electronic health records, medical records, personal health records, eHealth services for patients, patient portal, physicians, patient empowerment, patient participation, doctor-patient relationship

## Abstract

**Background:**

Roles in the doctor-patient relationship are changing and patient participation in health care is increasingly emphasized. Electronic health (eHealth) services such as patient accessible electronic health records (PAEHRs) have been implemented to support patient participation. Little is known about practical use of PAEHR and its effect on roles of doctors and patients.

**Objective:**

This qualitative study aimed to investigate how physicians view the idea of patient participation, in particular in relation to the PAEHR system. Hereby, the paper aims to contribute to a deeper understanding of physicians’ constructions of PAEHR, roles in the doctor-patient relationship, and levels and limits of involvement.

**Methods:**

A total of 12 semistructured interviews were conducted with physicians in different fields. Interviews were transcribed, translated, and a theoretically informed thematic analysis was performed.

**Results:**

Two important aspects were identified that are related to the doctor-patient relationship: *roles* and *involvement*. The physicians viewed their role as being the ones to take on the responsibility, determining treatment options, and to be someone who should be trusted. In relation to the patient’s role, lack of skills (technical or regarding medical jargon), motives to read, and patients’ characteristics were aspects identified in the interviews. Patients were often referred to as static entities disregarding their potential to develop skills and knowledge over time. *Involvement* captures aspects that support or hinder patients to take an active role in their care.

**Conclusions:**

Literature of at least two decades suggests an overall agreement that the paternalistic approach in health care is inappropriate, and a collaborative process with patients should be adopted. Although the physicians in this study stated that they, in principle, were in favor of patient participation, the analysis found little support in their descriptions of their daily practice that participation is actualized. As seen from the results, paternalistic practices are still present, even if professionals might not be aware of this. This can create a conflict between patients who strive to become more informed and their questions being interpreted as signs of critique and mistrust toward the physician. We thus believe that the full potential of PAEHRs is not reached yet and argue that the concept of patient empowerment is problematic as it triggers an interpretation of “power” in health care as a zero-sum, which is not helpful for the maintenance of the *relationship* between the actors. Patient involvement is often discussed merely in relation to decision making; however, this study emphasizes the need to include also sensemaking and learning activities. This would provide an alternative understanding of patients asking questions, not in terms of “monitoring the doctor” but to make sense of the situation.

## Introduction

### Patient Participation and Electronic Health Technologies

Patient participation is advocated as a means to improve patient safety and is seen as a key component in the redesign of health care. There are, however, many barriers to patient participation that according to Longtin et al can be divided into (1) patient-related and (2) health care professional factors [1]. Among health care professionals, the main obstacles to patient participation are the desire to maintain control, lack of time, personal beliefs, and insufficient training in the patient-caregiver relationship [1].

It is argued that electronic health (eHealth) technologies have a tremendous potential to promote patient participation and improve health outcomes [2]. eHealth interventions recently started to focus on patients’ rights to access their electronic health records (EHRs) over the Internet (eg, through patient portals). However, literature is not conclusive regarding the effects of making health records available for patients. Some studies have reported that patient accessible health records can generate anxiety or concerns [3], whereas others have concluded that having full access may decrease anxiety [4]. At the same time, it has been reported that health care professionals have been concerned about giving patients Web-based access to their health record (see eg, [5]). One of the concerns from physicians, as identified in a previous paper, was that patients would read their EHR with the purpose to control and monitor physicians [6]. Thus, they feared that patients would check on and monitor the physician’s activities rather than adhering to the more “traditional” relationship: that physicians check on the patient, and not the other way around. These results indicate that we need to further explore the doctor-patient relationship in relation to eHealth interventions aiming at increasing patient involvement.

The purpose of this paper was to analyze and report in depth about how the interviewed physicians view the idea of *patient participation* in general and in relation to patient accessible electronic health records (PAEHRs). This is important to understand how they make sense of and assess the introduction of PAEHRs and eventually to explore the possible relationship between their concerns and the patients’ abilities to become active partners in their care. The contribution of this paper is a deeper understanding regarding factors related to physicians’ framing of PAEHR in relation to patient participation. Furthermore, the paper contributes with a critical discussion of the concept of patient empowerment as being problematic as it triggers an interpretation of power as zero-sum.

In our previous paper [6] from this study, we gave an overview of our whole dataset, which was thematically analyzed in relation to the physician’s work environment. In this paper, we want to explore certain aspects of the data in more depth, which is in accordance to the method as presented by Braun and Clarke [7]. The detailed analysis conducted for this paper focused on patient participation and empowerment as an element of the patient-doctor relationship. The main research question driving this in-depth analysis was “How do physicians view the idea of patient participation in general and in particular in relation to patient accessible electronic health records (PAEHRs)?”

### The Patient Portal and PAEHR

Patient portals are provider-tethered applications that allow patients to access, but not to control, certain health care information (eg, their EHR) and provide communication and administrative functions (eg, secure messaging, appointment booking, and prescription refill requests) [8]. In 2012, Region Uppsala in Sweden launched a Web-based patient portal to its 350,000 citizens as part of a large European Union project. The portal offers about 10 different eHealth Web services and aims to contribute to patient participation. Efforts to enhance participation through eHealth solutions have been emphasized in the National eHealth strategy of Sweden [9]. The provided eHealth services include, for example, PAEHRs, including the latest test results, appointment booking, following a referral, and a list of names of all health care professionals who have entered the EHR (so called “log list”). The PAEHR captures information from different EHR systems in all of Sweden, and the patient can read information from the primary care as well as hospital care. What exactly is shown in the system depends on (1) the EHR system the provider uses and (2) the region where the provider is located and thus whether and how the PAEHR system has been implemented. It is not possible for patients to edit the records; however, in Region Uppsala, they can comment on each of the professionals’ notes. Patients access the portal using an e-ID or other secure log-in options. Initially, and at the time of the presented interview study, health care professionals had to sign or approve the medical notes for patients to access them within the first 2 weeks. This was later changed, in that patients in Uppsala now choose whether they only want to read signed notes or unsigned notes as well. Today, PAEHRs are provided in 19 out of 21 counties in Sweden and have more than 1,000,000 registered users [10].

### Doctor-Patient Relationship

Various models of the relationship between the physician and the patient have been discussed in literature (see eg, [11-13]). The different models have been developed over time in accordance with new approaches to conducting health care, for example, shared decision making [12,14] and patient-centered approaches [15].

In the following, we will briefly describe the basic models of the doctor-patient relationship by Szasz and Hollender [16] and the models discussed by Emanuel and Emanuel [12]. Even though the approaches to conceptualize the relationship overlap on certain levels (eg, they both include a paternalistic model), we consider them both relevant for our discussion as they emphasize different aspects. In Szasz and Hollender, primarily the role of the patient is changing (from infant to adolescent child to adult) [16], whereas Emanuel and Emanuel outline a spectrum of possible roles for the physician [12].

For each of their three basic models of the doctor-patient relationship, Szasz and Hollender describe the roles of physician and patient and relate the relationship to a prototype [16]:

Activity-Passivity: This is the oldest model, according to Szasz and Hollender, in which the physician “does something to the patient” [16] who is a passive recipient. This model is suggested applicable when the patient is unable to actively contribute (eg, acute trauma). This relationship is compared with that of a parent and an infant [16].

Guidance-Cooperation: Szasz and Hollender outline that this model is employed in situations in which the patient is conscious, and both the patient and the physician are active [16]. However, the patient’s activity is rather to cooperate and “obey,” as the authors put it [16], without questioning, arguing, or disagreeing with the physician’s orders. This is explained in that the patient places the physician in a position of power because the latter possess medical knowledge that the patient is lacking. The prototype of this model is the relationship between a parent and the (adolescent) child [16].

Mutual participation: In this model, the physician has the role to help the patient help himself, who as an equal partner in this relationship uses expert help [16]. Accordingly, the prototype of this model is the relationship between two adults. The authors explicitly refer to the management of chronic illnesses as clinical application, where “patient’s own experiences provide reliable and important clues for therapy” and where the treatment is often carried out by the patient [16].

The analogy of the relationship between physician-patient and parent-child has been made also by others. The very term of the *paternalistic model* already entails the reference to a “father.” Katz describes that patients may display childlikeness, which is “triggered not only by pain, fears, illness, and memories but also by how physicians view and respond to patients” [17]. Furthermore, by viewing them “too much as needy children, physicians disregard the fact that patients are adults as well” [17] who have certain needs such as wanting to be informed and involved.

In the four models of the “physician-patient relationship” outlined by Emanuel and Emanuel, the physician’s role varies between a *guardian* (paternalistic model), a *counselor or adviser* (interpretive model), a *friend or teacher* (deliberative model), and a *technical expert* (informative model) [12]. The paternalistic model, in which the physician determines what is best for the patient, leaves little room for the patient to participate [13] and should today merely be justified during emergencies [12]. In situations other than emergencies, patients’ participation is essential because although physicians might possess more medical knowledge, patients know more about their own needs [17]. In addition, some patients possess in-depth knowledge of their condition, which may even exceed that of the (more or less prepared) health care professional [18].

What emerges from the above is a quite disorderly view of what the doctor may or should represent to a patient and what kind of relationship between patient-doctor would need to be established. Solitary decision making by physicians has a long tradition in medicine and obscures the uncertainty of medical knowledge, which, as assumed by physicians, would lead to anxiety and confusion if brought to the patient’s attention [17].

### Patient Participation

As the changing roles in the doctor-patient relationship suggest, patient participation in health care, including decision making, is increasingly emphasized. Patient empowerment has been described as the attempt to increase the patient’s capacity to think critically and make autonomous, informed decisions [19]. Patient empowerment is surrounded by many other concepts such as engagement, enablement, participation, involvement, and activation [20]. In an attempt to clarify boundaries and relationships between these concepts, Fumagalli et al developed a concept map in which they combined the key definitions into “Patient empowerment is the acquisition of motivation (self-awareness and attitude through engagement) and ability (skills and knowledge through enablement) that patients might use to be involved or participate in decision-making, thus creating an opportunity for higher levels of power in their relationship with professionals” [20].

The question remains what involvement or participation in *decision making* means in practice. In what kind of decisions are patients involved and able to participate? Deber distinguishes between two dimensions of choice: *problem solving* (the search for the solution to a problem) and *decision making* (the choice being made from several alternatives) [21]. It has been noted that *problem-solving situations* require some level of medical knowledge and thus, do not present themselves well to patient participation, whereas certain *decision-making situations* require the patient to analyze and determine the value of potential outcomes [1]. This distinction is very relevant when it comes to the question whether patients “want to be involved” or would rather “leave it to the doctor to decide.” Research showed that patients are quite capable to discern between these situations [1,22]. In a study by Thompson, patients’ desire to be involved was much higher regarding decisions that do *not* require medical knowledge but that have lifestyle implications and where attitudes and values are likely to be important factors [22].

Similar to the doctor-patient relationship, different models of involvement and participation in consultation exist, which reflect various levels of patient power [23]. With an increasing level of patient power, the levels of professional-determined involvement are (0) exclusion, (1) information-giving, (2) consultation, (3) professional-as-agent, and (4) informed decision-making [23].

Despite a vast amount of research in this area, an in-depth understanding of the impact of PAEHR in relation to patient participation and the doctor-patient relationship is still lacking. To reach the aim to understand the physicians’ views of patient participation and PAEHRs’ possible effects on this, this paper adopted the models of the doctor-patient relationship and the various levels of involvement from previous research.

## Methods

### Interview Content and Data Collection

Semistructured interviews were conducted in the summer of 2013, about 6 months after the PAEHR service was launched. Twelve physicians were interviewed by three different researchers. All researchers used the same template for questions to cover the required areas of interest. The template consisted of 27 questions (see Multimedia Appendix 1) and was developed in cooperation through a number of meetings. All interviews were done face-to-face except one, which was carried out by email. On average the interviews lasted 1 hour.

### Participants

As reported in the previous paper [6], getting access to physicians who were willing to take part in an interview proved to be a greater obstacle than was anticipated. Different strategies were applied to find physicians, for example, contacting heads of departments and making use of mailing lists. The project nevertheless succeeded in getting a positive response from physicians in four different specialties: orthopedics, oncology, emergency medicine, and internal medicine. The characteristics of the interviewed physicians (N=12) can be found in Table 1.

### Analysis

All interviews were transcribed, translated, and repeatedly read by all authors. This paper is the second reporting of the study. For the first paper [6], a thematic analysis [24] was conducted in which the whole dataset was coded, also known as *complete coding* [7]. For this paper, a selection of data was used for an in-depth thematic analysis, which consisted of data that previously was coded “patient empowerment.” The selection comprised nearly 50 pages of interview excerpts, which again were thoroughly and repeatedly read through, jointly discussed, coded, and commented on.

The excerpts were printed to facilitate collation, clustering, and the development of a thematic map. The clustered extracts were read again for each theme to review the internal homogeneity [24]. Part of the analysis process was also the iterative development of a thematic map. The iterative process with several rereadings, discussions, and thematic descriptions was carried out with the aim to achieve trustworthiness in the research process. At the same time, as the potential themes were identified and reviewed, the authors read and discussed the wider literature to build on established concepts and in particular their distinctions (eg, patient-desired vs professional-determined involvement [23], overlapping meanings of concepts such as empowerment, engagement, enablement, participation, involvement, and activation [20]). The quotes used in this paper have been slightly edited to be more readable.

**Table 1:** An overview of the interviewees (N=12).

Characteristics		Number of interviewees
**Specialty, n**		
	Orthopedics (Ortho)	5
	Oncology (Onco)	3
	Emergency medicine (EM)	2
	Internal medicine (IM)	2
**Gender, n**		
	Female	5
	Male	7
Work experience (years), mean (range)		14 (2-30)

## Results

### Overarching Themes

**Figure 1:**
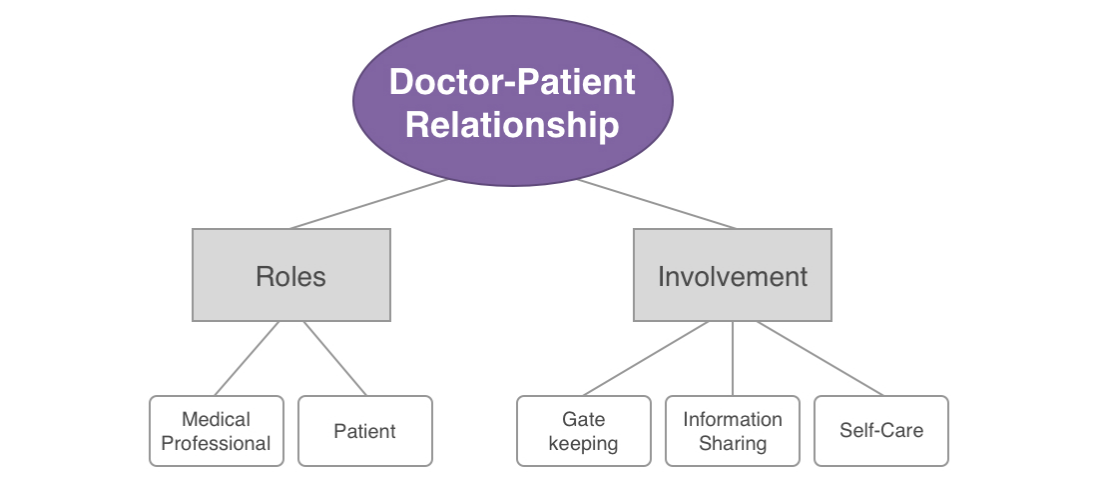
Identified themes from interviews with physicians related to patient participation.

The thematic analysis of the dataset resulted in the identification of the overarching theme *doctor-patient relationship* (Figure 1). The *doctor-patient relationship* captures two important aspects that were discussed during the interviews with physicians: the roles that are involved in this relationship (ie, the medical professional and the patient) and in what way patients can contribute in this relationship in terms of involvement (related to concepts such as gatekeeping, information sharing, and self-care).

In the following sections, the themes and subthemes will be described and discussed in relation to the wider literature.

### Roles: Medical Professional

The theme *Roles: medical professional* captures the way the physicians talk about their own role in relation to patient participation. When asked directly about patient participation and what this means to them and to their role, many physicians expressed that they were not generally opposed to this. They rather expressed that patient participation is important and that they are in favor of it. However, one common understanding of participation found in the interviews was that patients’ experiences need to be understood for the physician to assess them, and then the patient needs to be convinced about what treatment is the best, as exemplified in the following quote:

In order to get good treatment results it is really important for us to understand what problems the patient is experiencing so I know how I can respond to it and assess it. And then when we think we have understood what is wrong, then we need to discuss it with the patient, we must get the patient on board and make him or her also believe that this seems reasonable, that this is the problem.

EM-1

Moreover, on several occasions, the physicians indicated that for them patient participation is about presenting options from which the patients may choose. For example, when being asked what it would mean if the participation would increase, this physician responded:

I do not really think it [patients reading PAEHR] would mean so much difference at all, because already today you ask the patients how they want it, if you have two equivalent alternatives to present it is what the patient thinks that determines it.

Ortho-4

In our interpretation, the above presented ways of involving patients depicts the role of the physician akin to the *professional-as-agent*. According to Thompson, the professional-as-agent possesses “the technical expertise, but patient preferences are incorporated into their decision-making” [23]. As Thompson points out, the incorporation necessitates some prior dialogue and considers this as level 3 of patient involvement, which is the second highest level [23]. However, it can be discussed what this means in terms of patient participation, as in the quote from EM-1, the term getting the patient “on board” might be understood as getting the patient on board of the doctor’s boat and not the other way around.

Deber even argues that the values assigned to potential outcomes are not relevant to problem solving (ie, searching for the solution to a problem), and thus, patient participation is not necessary to identify alternatives and to estimate their possible outcome [21]. However, if the preferences and values do *not* play a role and the physicians determine the “equivalent” alternatives *without* a dialogue, we claim that this cannot be considered as patients participating in decision making.

Some physicians claim that the physicians need to have the final say when deciding upon a treatment and that there is a limit to patient participation in relation to science, as exemplified in this quote:

There is some sort of limit to how much should the patient [be involved]. Where is the line between being involved and to decide? We do have some treatments that we claim to be better than others, but some patients come with a belief that, well, they have read or heard that a friend got this other treatment and think it is better, and /.../ I think that the doctor should have the final say in the case if you are basing your opinion on science.

Ortho-3

This comment shows the opinion that evidence-based decisions should be prioritized. According to Williamson, ethical challenges are raised when no *clear* evidence-based solutions are available [25]. In these cases, the decisions have to be supported by patient’s values and preferences. However, few physicians in our interviews acknowledged the ethical challenges. More often it was a matter of “convincing” the patient of the best treatment options regardless of individual preferences. We interpret this way of making decisions as an example representing the physician in a paternalistic role.

Another aspect of the medical professional’s role was the question of responsibility and trust. This was discussed in relation to patients accessing their EHR. Taking care of patients was seen as the physician’s responsibility. As long as people trust their physician, there should be no need for patients to read their records, according to some of the physicians. Most physicians also believed that the patients already know what they need to know without reading their EHR, as stated in the following quotes:

We are trying to do the best for all patients. We are the ones who take responsibility for complications and everything so they're going to try to enjoy life and not sit in front of a computer and check test results and devote time to it.

Onco-1

I believe that most patients feel that they know what they need to know, and have the influence according to their level of knowledge, or how to put it. It’s a bit like if I leave the car to the mechanic, I do not expect that I’ll know exactly what they will do, but I’m happy if they fix it—sort of.

Ortho-2

Furthermore, patients who intend to be more involved in terms of asking questions were interpreted by one physician as a sign of mistrust. This mistrust was also seen as a recent phenomenon related to physicians’ status in society and a lack of respect for the physician’s high education. One physician stated as follows:

And there is a mistrust in this that bothers me very, very much. A mistrust for what I suggest. /.../ What has happened to the old image of the doctor who was very good and “now you are going to meet the doctor” and it was a person with a high status. I don’t have to be seen as some demigod, but I want to be respected for the education that I have. It takes years to become a doctor, and even more years to become an orthopaedic, and all these years of education now count as nothing, because the patient should choose now. And that mistrust bothers me. Do I feel that mistrust, then I usually say to the patient that this will not be good. I will transfer you to another doctor.

Ortho-1

We interpret that the idea that patients should give physicians full responsibility and trust them to know what is best for the patient is closer related to paternalism than to a partnership in care. In paternalism, “the professional knows best and patient involvement is limited to being given information or giving consent” [23]. In summary, the way the physicians talk about their role emphasizes that they are supposed to take on the responsibility, determine the alternative options from which—if equivalent—the patient may choose from, and the patient as such should not be involved in this process but only trust the physician’s judgment. In this sense, there is no risk of “abandoning the patient,” which was emphasized as being a risk when the autonomy is too high (compare to [25]). This high level of autonomy would correspond to involvement level 4—informed decision making—where the patient makes the final decision after the technical expertise is transferred to him or her [23]. However, we would argue that in the physician’s construction outlined previously, the patient is still left with a rather passive role, which is not in line with attempts to establish a partnership among equals, nor in line with the idea that patients should read their EHR.

### Roles: Patient

The theme *Roles: patient* captures the way physicians talked about the patients in terms of their skills (or lack thereof), motives to read the EHR, and **c**haracteristics.

#### Skills

The physicians discussed certain skills that are needed, but which patients might lack, to *benefit* from accessing their EHR. For elderly people, some physicians assumed that they do not have that skill as they are not that familiar with the technology needed. Thus, according to physicians, elderly people are probably not that interested in reading their EHR, which is illustrated in this quote:

I most care for the elderly and then, they usually do not have an interest. They need to have e-identification, for example, and Internet and stuff, and they usually don’t have that. So, actually it is more relatives like me, who want medical record copies, but not the patient himself/herself.

IM-2

Although the assumed lack of technological skills was related to elderly adults only, the lack of medical knowledge was discussed in relation to all patients. Because the content of the EHR must contain medical terms, it was assumed that patients would not understand it or even misunderstand its content. One physician stated the following:

The record is not a means of communication with the patient, the record is a tool and therefore it must contain medical terms that the layman does not understand for it to be an effective communication tool among doctors and other health professionals.

Ortho-2

In relation to the content, it was not only the jargon that was assumed to be difficult to understand by the patients but also to determine which information is *relevant* or *important*. Almost all physicians discussed the complexity of the records and that they are difficult to interpret and evaluate (even for them). The records must therefore be filtered and interpreted for the patients to help them understand. One example often mentioned by the physicians in the interviews was what the particular lab results mean for patients. Without the physician’s guidance and support, patients would focus on details and not understand the real meaning of lab results. Moreover, physicians claimed that patients refer to details in the record that are of no importance to the physicians, as exemplified by the following quotes:

There are also a lot [of cases] where a lab value or something is outside the reference range but when it does not mean anything, which I think the average citizen does not understand: “But it is outside the reference range, so surely something must be wrong.”

Ortho-2

And then I have had patients who several times have printed their record and then they say: “You write here that I had pains in two weeks, but the truth is that I did have pain for three weeks.” And then they talk about bitty details in the record and talk about all the details.

Ortho-1

These comments exemplify that the professionals regarded certain aspects or wordings as not as important or relevant as their patients did. Whether the particularities in the records (2 vs 3 weeks as in the quote above from Ortho-1) are relevant might be questioned. It is understandable though that patients are interested in having errors corrected, which has also been reported by Esch et al [26]. However, Rexhepi et al reported that few patients actually ask for errors to be corrected [4]. In addition, we are faced with the question of whether the patient is static or not. Related to the view of patients not being able to understand lab values, patients may learn over time to interpret the results, especially if the same tests are taken on a regular basis.

One way for physicians to handle that patients have difficulties interpreting the information has previously, before PAEHR, been to channel and select the pieces of information they give to the patient. This strategy, however, is now challenged when patients can access their record and tests results directly. This change is illustrated in the following quote:

If, for example, you are examining a patient, you don’t give them all answers one by one as they come in. Instead you make a plan and decide how the patient is and what sort of information they can take. Then it is difficult that they can just go and read it all by themselves without having any idea what that means mostly, really, so it's not good.

IM-2

We interpret that this way of presenting patients with selected information is a type of interaction that follows on the paternalistic model of doctor-patient interaction [12]. This model describes how the physician provides the patient with “selected information” to “encourage the patient to consent” [12].

The physicians were aware of, however, that today patients are also looking on the Web for further information. In the interviews, this was regarded as both something positive and negative. Although searching on the Web might clarify questions for patients and increase their knowledge, some physicians were concerned that patients might not be equipped to interpret all this information, as exemplified by the following quotes:

They sort of clarify, sort out the question marks. [Using Internet resources] may provide tools to find out more. If I say that they have the disease X, they can go home and google or go to the library or whatever they do to acquire knowledge and learn more about it.

Ortho-3

You can search [online] and many do, both patients and parents. So, you go out and search Google for various treatments. And seek their own information, and there is the problem to be able to evaluate the information you find because a lot is not scientific or quality assured.

Ortho-4

There is nothing that is worse than a patient that has read things on the Internet and says “I absolutely do not have heel spur.” Then I say “you do have heel spur.” Then they go: “I read on the Internet and I do not have heel spur. I read on the Internet and don’t have everything that it says there.” But then I say “that the other 19 things on the list out of 20 was correct, so you have heel spur. From experience, I know that if you have 19 out of 20 then you do have heel spur.” “Well, that one was not correct.” I have these problems all the time, and it is just because the Internet exists. Very tough and energy consuming! This is because Internet has no control. Internet contains anything at all. If you enter the wrong page, and read the wrong thing where someone who is not serious writes, then you can get the wrong information.

Ortho-1

#### Motives

During the interviews, the physicians mentioned some possible motives to (or the lack thereof) why patients might want to read their EHR. Lacking interest in reading was connected to elderly adults who are satisfied with what they know. The elderly do not “want to know everything,” according to physicians. If a physician perceives that patients today put less trust in the role of the doctor (see above), this was assumed to be a motive for the patient to read the EHR. Thus, some possible motives to read were negatively associated by the physicians, for example, regarding patients to act as the police or someone who check on the doctor. One physician stated the following:

I am very afraid of misunderstandings. Misunderstandings, and mistrust, and some already say that. Do you [the patient] think that I do what is worse for you, or what do people think? Why do they need to check me? If you have that perspective and there is a misunderstanding, then everything can happen. SO WRONG!

Ortho-1

A few physicians mentioned that some patients need their health records for an insurance claim or because they want to share the record with family members who work in health care. Several motives in relation to a medical interest were also mentioned: patients can have an eye on the progress on the Web as well, use the EHR as a memory aid about what has been discussed during the visit, or read the test results before their next planned visit.

#### Characteristics

**Figure 2:**
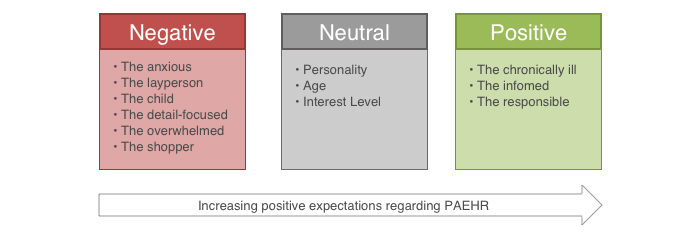
Characteristics of patients that were associated with negative, neutral, positive expectations toward patient accessible electronic health records (PAEHRs).

The physicians’ expectations ranged from rather negative to neutral to rather positive in relation to patients accessing their EHR (Figure 2). A negative or a positive expectation (red or green box in Figure 2) could be related to a certain stereotype of a patient, which physicians used as examples. Where the expectations were rather neutral, the physicians would not describe a specific “type,” but instead they discussed the possible characteristics on which the outcome might *depend* (grey box in Figure 2) such as personality, age, or whether they are interested in the first place.

If physicians were expecting rather *negative* behavior from patients accessing their EHR, patients were described as anxious, detail-focused, overwhelmed (ie, who need guidance), would possibly “shop around,” or as laypersons. These types of patients would demand that the physician either would need to calm the patient, to guide him or her, or to explain again the situation.

This extra work would arise because of the existence of PAEHRs, according to these physicians. In addition, certain activities (eg, guiding the patient through their results) would be impeded when patients read their records on their own.

In some instances, physicians commented on the patient’s access in a rather *neutral* way, while acknowledging that certain aspects depend on the patient. For example, whether patients are involved directly would depend on their personality, their general interest, or their age (ie, the elderly are perceived as less likely to be actively involved or to have Internet access). Thus, in these instances the physicians did not have fixed expectations and did not describe a specific type of patient.

Few physicians commented in a *positive* way about certain patients accessing their EHR and thus, possibly benefiting from this eHealth service. Here, the physicians described certain types of patients, namely those who are chronically ill and thus “extremely interested in their healthcare” [Ortho-5] or patients who are well-informed and read up. A last group of patients was described as taking responsibility for possible consequences of their actions (eg, if they read their record outside of office hours and thus face the consequence that they cannot immediately contact health care to ask questions).

It is interesting, that none of these physicians discussed how a patient could possibly develop from the left category (negative) into the middle (neutral) or right category (positive). People are not a static entity but are able to develop skills and knowledge over time. It could well be that the chronically ill patients and those who are well-informed and read up were not always like this but developed over time. However, even well-informed patients might face new situations where they lack knowledge, have to adapt, and start a new learning process.

One explanation could be that the interviewed physicians from the hospital might not treat the individual patient on several occasions. In other words, lacking a continuity of care makes it difficult for physicians to develop a long-term relationship with a patient and thus, recognize the potential developments.

### Involvement: Gatekeeping

*Involvement* captures aspects that support or hinder patients to contribute and take an active role in the doctor-patient relationship. Involvement includes themes such as gatekeeping, information sharing, and self-care.

The interview questions centered around the patient portal in general and in particular in relation to the eHealth service, which gives patients access to their EHRs. It is thus not surprising that many aspects that touched upon involvement of patients in their care were related to information sharing between doctor and patient. One of the introductory questions, however, explicitly addressed physicians’ thoughts on patient involvement and what it could mean for them if the participation would increase.

Some physicians were negative to patients reading their EHR by themselves and would rather prefer physicians to be the contact person for patients, as exemplified by the following quotes:

Doctors are honest and are telling the truth, why should they [patients] get access to the record, they can surely come to visit and discuss possibilities and explanations.

Onco-1

It is not that we want to hide something or, but there is a world of its own and we have gone through a long training to handle it in the best way and then you have to also let us do the work without checking everything and interfering.

IM-2

I think it’s inhumane to patients, who of course then go in [the EHR] and look, because they want to know, and they think “I can handle it.”

Onco-3

As aforementioned, at the time of the interviews, patients could only see those records that were signed by the physicians or were older than 2 weeks. The necessity to sign notes could at this time be used by physicians to prevent patients from accessing information as is described in the following quote from an oncologist:

I will no longer sign test results in the same way as I did. If there is any progress then I will not sign since the patient can go in and read the answer before I have had time to call and tell them.

Onco-1

The same physician also considered writing more vague descriptions and wait with the specific details until they were discussed with the patient. Both, changing the way of writing and thus limiting the information provided and not signing the notes to prevent immediate access, can be interpreted as “covert ways to remain in power,” as described by Longtin et al [1].

### Involvement: Information Sharing

In this section, bilateral information sharing between the health care system or physician and the patient is discussed. On the one hand, the health care system is sharing information with the patient by giving them access to their EHR through the patient portal. On the other hand, patients are also sharing information by *not* blocking parts of their record. Patients are entitled by law to block certain parts or all of their medical records and then the records would not be accessible for health care professionals unless there is an emergency situation. The interviews addressed also the prospect of patients blocking information; thus, the discussion also considered information that is shared (or not shared) with physicians.

Physicians were not critical about giving information to patients in general, on the contrary, they considered this as important, as exemplified by the following quotes:

The patient must be well informed about their disease and what we might plan to do, what are the opportunities, what the prognosis is, treatments, that they will have an influence over what they will be going through, and for them to be involved so they must have been properly informed.

Ortho-2

It is very, very important that we give information why we do different things, what is happening around you. “Why should I not eat, why should I shower with a special soap?” /…/ Before the doctor told the patient what to do and the patient did it without really understanding, and nowadays that does not happen. Today people require to understand why and I think that is reasonable.

Ortho-1

In the quotes above, the physicians talk about the necessity for patients to understand what is happening around them and exemplify this with the reasons behind certain *instructions* that the patient is supposed to follow. However, patients may ask questions about aspects that the physician may not deem to be relevant at that time. One physician stated the following:

Or they bring a record that some other doctor has written, and then they say that “I don’t understand these words. Could you explain them to me?” Then I have to take time from my schedule to explain this to them. When it wasn’t even me that had written them to start with.

Ortho-1

Although these kinds of questions may be perceived as a burden, we believe that they might nevertheless help the patient to make sense of the situation. This sensemaking may in turn be necessary to be able to ask further questions to reach an understanding.

On several occasions, the physicians mentioned that the patient has the right to see the information; even though some were critical about *when* and *how* this information was shared. One physician stated the following:

The patient has the right to know /.../, they have the right to read the record and get to know how we have written about it, and it's not something secret, I mean we are talking to the patient about what is happening and we explain everything.

Onco-1

Some physicians reflected on how information is shared with the patient in general (ie, without information and communications technology [ICT]) and that this process is also not perfect, as exemplified by the following quotes:

I sometimes think that there is a certain lack of written information about various diseases because, uh, at a clinic visit, the patient is often stressed and /.../ it is very much information to take in, uh, so I think that many would have needed to read something a bit more structured after the visits, for example.

Ortho-2

It is difficult as well to memorize everything that is done and all samples in a clinic visit, so now you can sit at home in peace and quiet and read the record.

Ortho-5

If you put demands on the patient to rest their foot and stay in bed for two weeks, then they need to understand why they need to do that. /…/ We are not very good at giving that information, and that information should be on the Internet. There is where people look today.

Ortho-1

The responses indicate opportunities for ICT to improve information sharing with the patients, for example, by giving them more structured information. However, it was strongly questioned whether giving patients access to their EHR would solve this lack of information, as it was assumed that this would worry patients, and they would not understand more from what they have read. In the quote below, Ortho-1 expresses that there are better ways to inform patients than to give them access to their EHR:

I think that you solve the wrong problem. Patients are not informed; they feel non-informed. And I agree with that. Worried, they don't understand, they are not doctors themselves. This is solved by them reading their medical record? It is the wrong solution to the problem! Then they become more worried, and more upset, and don’t understand. All this worry should be met in a better way with better information and better general information. Better information about what will happen during your surgery, what does this word mean, what will happen now.

Ortho-1

Physicians sharing specific information with patients can be interpreted as level 1 of patient involvement, which includes “simply giving them information considered necessary by professionals” [23]. Considering that level 0 is actually the level of *excluding* patients [23], we regard the level of involvement as not very advanced on level 1. Thus, although physicians were positive toward sharing information on a more general level, there is room for improvement on the way to a partnership. In particular, information sharing should possibly go beyond what professionals consider necessary and also reflect the patient’s need for information.

Most physicians were, however, critical about patients’ possibility to block certain information in their EHR and by this, denying the professionals access. Although few agreed that there might be instances where the patient might want to block sensitive data, they considered this as a potential threat to giving patients proper care. However, most physicians acknowledged that patients might want to exercise their right to block information but would then also have to take responsibility for it, as exemplified in the quote below:

Basically, it’s right that you determine the course of your own body and what others should know about it. But on the other hand, I fear that it might get out of control and that it can eventually become a danger to a patient and information which for me can be very important that I cannot access can then lead to me giving wrong or even dangerous care to someone. I can understand it, but for my treatment of the patient it is a risk that it is something bad. So overall, I think it’s unfortunate, but at the same time, it is perhaps something you have to accept that people decide over themselves.

Ortho-3

It was seen positively, however, if the system would indicate whether anything was blocked, so that they could engage in a conversation with a patient on whether they want to share this information with them, as expressed by EM-1 in the quote below:

That would be a bit better. Then I can see that something is hidden, and I can ask the patient: “Something has been blocked here, would you like to tell me what is says there?” And you might be able to solve it that way. If this is the way they have chosen to go, this might be a better solution.

EM-1

The physician’s concerns of patients blocking them from information is interesting, in that it can be related to the idea that only the professionals are in the position to determine whether a piece of information is relevant or not. This refers not only to what should be shared with the patient but also what information should be available to the professional and what kind of questions is relevant to discuss. It seems that physicians have the motto “the more information the better” when it comes to themselves and their work. However, the same motto does not seem to apply to patients, as it is assumed they would be overwhelmed and focus on irrelevant details.

### Involvement: Self-Care

The theme *self-care* captures discussions around patients who have the ability to care for themselves and perform activities necessary to achieve, maintain, or promote optimal health, as has been defined by Richard and Shea ([27], as cited in [28]). Although the physicians tended to discuss their own responsibility in taking care of the patients, on few occasions it was mentioned that patients can also engage in self-care and might benefit from technological support. The quote from EM-1 is one such example:

If you think of the big picture, I think it is a lot down to the available information, that the patient is worried about some disease and they can get information from reliable sources, not just googling for it, get help to make their own judgment about what they believe so that they may be able to engage in self-care or they could actually wait a few days and make an appointment at the local surgery, they do not need to come to the emergency ward. Then they are more involved in their own healthcare

EM-1

As discussed previously, only few physicians mentioned patients with chronic conditions, although these patients often engage in self-care and perform many health-related tasks outside the doctor’s office. Although some physicians talked about this patient group and the potential benefits for them to access their EHR, others considered this group as rather marginal, as exemplified in the quote below:

There could possibly be some chronically ill patient who is extremely interested in their healthcare and who review and evaluate the information in their medical records. They would possibly be able to control their disease better, but it is such a very small group, and the group gets this information in any case. One can only ask that you be kind and print test results on paper when you’re at the doctor or whatever it may be, you can print the X-ray and so on. So, I do not think it will change, the possibility has been for the small, small group it concerns.

Ortho-5

Patients being encouraged to change their behavior can also be related to self-care, in that they have to take action themselves. The following comment exemplifies the idea that to emphasize the need for a behavior change, physicians might use the EHR to add comments for the patients to read, for instance “quit smoking,” as exemplified in the quote below:

But it’s a difference if it comes to, for example, smoking cessation, then you need a lot of participation that the patient himself realizes: “oh well, I also have to do something.” You try to help the patient get medical care or a group or stuff like that, then we can try to help the patients to make the step themselves.

IM-2

A few physicians discussed the possibility that patients could use their EHR as a memory aid to review instructions, which may then lead them to better follow those, as exemplified in the following quote:

It would surely be that the patient gets easier opportunity to review what was said at such a clinic visit, to remember more all instructions maybe, or together with the information you had, because you know that it is difficult to absorb all the information during a visit so, so that it is clear that there can be an advantage to have it as repetition.

Ortho-2

It has been emphasized that patient empowerment is the “antithesis of compliance” [19], in that empowerment-based interventions help patients to “think critically and make informed decisions” [19]. Thus, given this view, reaching a better compliance should not be the main objective for giving patients access to their records. However, reviewing the information discussed during a visit can support patients’ self-management, which in person-centered care is “another route toward greater participation” [25].

## Discussion

### Limitations

A limitation inherent in using interviews as a method for data collection is that what participants report may differ from what they actually do. In addition, some statements in the interviews related to patients reading their EHR were rather expectations than actual experiences. For the coders, it was partly unclear whether the participant reported on an actual experience. However, the analysis was focused on the descriptions of their daily practices and how they explained their concerns in depth and related the constructions to concepts found in the wider literature (eg, models of doctor-patient relationship).

As the interviews took place only a few months after the launch of the system, it is possible that attitudes might have changed. Although a survey conducted in another region in Sweden (Region Skåne) about 2 years after the introduction of the service suggests that this might not be the case [29]; follow-up research in Region Uppsala is needed and already in the works.

As the interviewed physicians had different specialties and worked at the hospital where a continuity of care might be lacking, results may be different with general practitioners (GPs) or physicians who are able to develop a long-term relationship with their patients. In addition, limits of participation as seen by physicians may differ whether they are specialists (eg, oncologists or orthopedists) or a GP. Further research is needed to investigate whether the different types of the relationship (eg, short term or long term) or the gravity of information or decisions they are dealing with influences the physicians’ attitude toward patient participation.

### Conclusions

The conclusions are related to two areas: (1) the doctor-patient relationship and the possibilities to use PAEHR as a tool for patient participation and (2) the use of “patient empowerment” as a problematic concept.

#### The Doctor-Patient Relationship and PAEHR as a Tool for Patient Participation

Already in the nineties, it was argued that unless there is an emergency situation, the paternalistic model of a doctor-patient relationship is not beneficial. The main reason is that this model assumes that patients and physicians share similar values and views, which is an assumption that may be incorrect [12]. Instead, shared decision making as a collaborative process in the medical encounter has been advocated [12,14]. Although the physicians in this study answered that they, in principle, were in favor of patient participation, the analysis found little support in their descriptions of their daily practice that participation is actualized. On the contrary, there were several signs of paternalism. This interpretation was further strengthened by the expectations that physicians often held regarding the characteristics of their patients (eg, as being unable to understand and as being worried and anxious).

The paternalistic model was also mirrored in the interviews, in that physicians described that patients only should be provided with pieces of information that they might be able to take in at a certain point in time. The physicians reacted strongly when patients could read the results before they had finished their process (ie, the investigations). In addition, physicians also criticized that they would have to spend extra time explaining and/or discussing information patients found themselves, which they would not have to do otherwise. It was also mentioned repeatedly that patients should trust the physicians, who are not working against the needs of the patients but have the best for them in mind. This exemplifies an assumption of shared objectives, which is an important dimension of the paternalistic model.

At the same time, physicians tended to view patients’ questions as signs of critique and mistrust. In our interpretation, this is an interesting struggle between the need for a “patient that understands” and the negative reactions that questions may evoke. We defend the possibility for patients to pose questions not only regarding instructions but also regarding other “objective facts” because to the patient, this is a process of sensemaking that is not necessarily of the same kind as to the professional. Making it visible for physicians which of their patients read their EHR might alter the doctor-patient meeting. Knowledge of whether the patient has read their EHR might help physicians to open up the discussion and hereby, create an open atmosphere where patients feel comfortable to talk about what was said in the record. This could be an opportunity to increase patient involvement to higher levels than was expressed in the interviews, which we attributed to level 1 (information giving) [23]. However, making it visible to the physicians who interpret patients reading as a sign of mistrust might affect patients so that they read less.

Low health literacy and the lack of knowledge of the subject is one obstacle to patient participation [1]. Although it still is the case that some patients do not want to be involved more than necessary, we believe that by opening up and inviting patients to ask questions, they will be more willing to participate, which could improve their health literacy. Considering that people develop skills and knowledge over time, reading their own record through a patient portal might enable patients to ask more questions.

As seen from the results, paternalistic practices are still present even if professionals might not be aware of this. PAEHRs make it much easier for patients to have access to their data without having to ask for permission. Even if the professional motto “the more information the better” could not be realistically applied to a patient, by the support of PAEHR we can at least avoid the patient being caught in a restricted situation of “less is more.”

We see PAEHR as a tool that opens a path for patients in that it is more difficult for professionals to exclude them. This is, however, only the first step. Our analysis highlights a clash between the principles underlying physicians’ practices and PAEHR, namely paternalism versus participation. This is an important finding. We believe the potential of PAEHRs is not reached yet, in that they could support not only the communication but might also support a change of the doctor-patient relationship toward one among equals.

#### “Patient Empowerment” Is a Problematic Concept in Academia and in Practice

Following from our analysis, we believe that a concept like “patient empowerment” is not helpful, in that it implies a loss of power and control on the part of the professionals. Health care is traditionally hierarchical and, especially in the paternalistic model, professionals have the authority. Emphasizing the *empowerment* of patients triggers an interpretation of “power” in health care as a zero-sum, meaning that a gain of power for one side (ie, the patient) entails a corresponding loss for the other side (ie, the professional). Although we are not opposed to giving patients more power, we consider this interpretation as not helpful for the maintenance of the *relationship* between both actors. In addition, we do not consider power in health care as a zero-sum situation in which the patient in the end will “take over” from the professional who ends up being a mere the technician who gives an advice (compared to the “engineering model”, [13]). We perceive, however, that patients might have other reasons to follow up on the test results than the professionals might have. Although professionals read the information in the record to diagnose the patient and plan the treatment, the patient might feel the need to read the record to follow up on what is happening (eg, reading the log list to check whether anything is happening), to start a sensemaking process, to prepare for the next meeting, or maybe even to process what is happening to them. The patient empowerment definition by Fumagalli et al includes the acquisition of motivation and ability to be involved; however, it focuses merely on *decision making* [20]. Relating to the above discussion, we prefer a term like *participation* over *empowerment* and would extend this to include also sensemaking and learning activities. Sensemaking and learning are ongoing activities throughout the health care pathway. Even patients with chronic conditions encounter new situations through new symptoms or relapse, in which the sensemaking and learning process might start yet again.

A commonality between the presented models of the doctor-patient relationship in the background of this paper [12,16] is that patients are described as being static and unchanged. However, patients can develop over time from an inexperienced “childlike” patient into an “expert” patient or the other way around. Albeit not easy, professional attitudes and practices have to be reexamined, including the view of patients as static and unchanged. Other things that need to be reconsidered are the importance of hiding uncertainties from patients, the need to appear authoritative, and to view patients as not sufficiently competent to participate in decision making (compare to [17]). This can be related to health care professionals who have to “unlearn being in control” when patients are becoming more empowered [30].

In this collaborative setting, PAEHRs can contribute to the development in the doctor-patient relationship, in that it opens a way for patients to play an active role and makes it more difficult for physicians to maintain a strategy that potentially exclude patients. Thus, it is likely that the relationship between doctor and patient is changing; however, the question is *when* a transformation will take place and *how* the relationship will develop over time. One might hope that by being able to read one’s records, the involvement increases and hereby, the distance between patient and physician decreases toward a relationship among equals. However, there is a risk that the future still remains “the silent world of doctor and patient” [17], where professionals do not ask and patients do not tell whether they read their records. Further research is needed on how patients make use of the record and whether professionals are today still unaware whether their patients read. In addition, the question is also how both actors may be able to support each other. For instance, professionals may be able to support patients to make sense of what is currently happening to them, and patients can help professionals understand what they are dealing with on a day-to-day basis. In that sense, we believe that eHealth does not need to be a “power struggle” in the doctor-patient relationship but can potentially help both partners to improve their relationship collectively and to grow individually.

## Appendix

Multimedia Appendix 1 Interview template.

## Abbreviations

eHealth: electronic health
EHR: electronic health record
GP: general practitioner
PAEHR: patient accessible electronic health record
